# Maternal Attitude and Knowledge Regarding Painless Labor: A Report from a Referral Hospital in Northern Iran

**DOI:** 10.5812/aapm-139079

**Published:** 2023-10-08

**Authors:** Fatemeh Hosseinzadeh, Zahra Hamidi Madani, Reyhaneh Shahrokhi Rad, Soheil Soltanipour, Zahra Rafiei Sorouri, Gelareh Biazar, Zahra Bagheri

**Affiliations:** 1 Department of Obstetrics & Gynecology, Reproductive Health Research Center, Alzahra Hospital, School of Medicine, Guilan University of Medical Sciences, Rasht, Iran; 2 Department of Anesthesiology, Anesthesiology Research Center, Alzahra hospital, Guilan University of Medical Sciences, Rasht, Iran; 3 Department of Community Medicine, School of Medicine, Guilan University of Medical Sciences, Rasht, Iran; 4 Student Research Committee, School of Medicine, Guilan University of Medical Sciences, Rasht, Iran

**Keywords:** Pregnant Women, Knowledge, Attitude, Painless Labor

## Abstract

**Background:**

Pregnant women’s knowledge about labor analgesia and the acceptance rate of this method are still undesirable in developing countries.

**Objectives:**

This study aimed to examine pregnant women’s knowledge, attitude, and acceptance of painless labor.

**Methods:**

The present observational study was conducted in a referral university hospital in Northern Iran from September 2022 to April 2023. Eligible women were interviewed; the data were analyzed in SPSS v. 22 and expressed in numbers and percentages. A P-value < 0.05 was considered significant.

**Results:**

The data from 369 eligible women with an average age of 30.39 ± 5.42 years were analyzed. Of these women, 7.6% had minimal information about labor analgesia, and 92.4% declared they were almost aware of the procedure. Only 6 women (1.8%) believed that the anesthesiologists were responsible for performing labor analgesia, while 218 (63.9%) considered it the duty of obstetricians-gynecologists. Besides, 294 women (86.2%) requested this method, and 259 (76%) were ready to pay for it. Moreover, 166(48.7%) had no fear of the procedure. Nonpharmacologic methods were the first choice for 164 (48.1%), while Entonox was the last choice for 26 (7.6%). A significant association was observed between maternal level of education and willingness to pay for painless delivery (P = 0.006), knowledge of who performs it (P = 0.015), requesting a painless delivery (P = 0.0001), options related to the preferred method for painless delivery (P = 0.001), and being ready to pay for a painless delivery service (P = 0.0001).

**Conclusions:**

Despite the poor maternal knowledge regarding the process of painless labor, the majority of the women requested the method and were ready to pay for it. These promising findings encourage the application of practical strategies to remove barriers.

## 1. Background

Painless labor (PL) took place in Paris in 1952 for the first time. However, it was abandoned due to the civil society’s indifference to it and the lack of political will. Before that time, it was believed that the pain of childbirth was the price of sin, according to the Bible (1). Painless labor is defined as relieving pain during childbirth via pharmacological and nonpharmacological methods with different degrees of success (2-4). Studies showed that the fear of pain is the main reason for women’s rejecting normal vaginal delivery (NVD) and choosing cesarean section (CS) (5). Women undoubtedly have the right not to suffer from pain and have a stress-free labor. The adverse effects of moderate-to-severe pain, such as sympatric release, unstable hemodynamic status, and the onset of the inflammation process, are well-known (6). Supporting painless delivery is an essential step, given the several advantages of NVD compared to CS. Pain management leads to the mother’s satisfaction and a better mother-infant relationship (7). However, as with other medical interventions, informed consent and patient acceptance are the first steps. In developed countries, the importance of PL has been confirmed, and research has focused on finding the optimal options in terms of cost, safety, and ease; however, in underdeveloped areas, maternal knowledge and even awareness of the possibility of PL are not investigated (8). Limited studies have explored this issue in Iran; besides, due to cultural, religious, and belief differences, the findings of these studies cannot be generalized, and each area should explore its actual conditions (9).

## 2. Objectives

This study aimed to investigate pregnant women’s attitudes and knowledge regarding PL. Since our hospital is a university referral center with almost 6,000 deliveries annually, the results of this paper could prove helpful for health policy-makers.

## 3. Methods

After the approval of the Research Ethics Committee of Guilan University of Medical Sciences (GUMS), this cross-sectional descriptive study was conducted at the Obstetrics & Gynecology ward.

### 3.1. Inclusion Criteria

Pregnant women who were admitted to the labor ward of Al-Zahra Hospital, were willing to participate, and gave informed consent were included.

### 3.2. Exclusion criteria

Pregnant women who were scheduled for CS due to specific indications, in the first or second trimester of pregnancy, unable to communicate properly due to speaking a different language or other reasons, or unwilling to participate were excluded.

The evaluation instrument was a questionnaire adopted from Sami Hassan’s study, translated into Persian, and approved by 10 expert faculty members. A face-to-face interview direct interview was conducted before delivery in a quiet place in the labor ward.

The first part of the questionnaire was about maternal demographic data such as age, employment status, parity status, residency, and education level. The previous mode of delivery and pain intensity during the last NVD were also asked. The second part contained questions regarding maternal knowledge and attitude toward PL. A pilot study was conducted on 10% of the calculated sample size to examine the feasibility and clarity of the questions. It was found that filling out each questionnaire took approximately 10 - 15 minutes, and all the items were meaningful. Therefore, the questionnaires completed during the pilot study were not excluded.

### 3.3. Sample Size

According to M Workie et al. (10) and Mung’ayi et al. (11), awareness from PL was 32%. Considering a 95% confidence interval, 5% error, and 10% nonresponse rate, a sample size of 369 women was estimated.

### 3.4. Statistical Analysis

The collected data were analyzed in SPSS v. 22 (IBM Corp., Armonk, NY, USA). The data were described by mean, standard deviation (SD), and frequency. Two independent *t*-tests were used to analyze continuous quantitative data in case of a normal distribution. In case of a non-normal distribution, the equivalent nonparametric test was used. We used the chi-square test for the nominal qualitative data. The significance level for all the tests was P-value < 0.05.

## 4. Results

Overall, 643 pregnant women were invited to participate. Among them, 26 disagreed to be interviewed, 14 had communication problems, and 234 had not heard of PL; as a result, the next questions were not asked. Finally, the data from 369 eligible women with a mean age of 30.39 ± 5.42 years were analyzed. Table 1 presents the maternal demographic data. Of the included women, 7.6% had at least information, and they had only heard about the availability of this opportunity without further information, and 92.4% declared they were aware of the procedure. The primary source of their information was the internet (n = 170, 42%), followed by friends and relatives (n = 123, 30.4%). Only 6 (1.8%) were aware that anesthesiologists were responsible for performing PL, while 218 (63.9%) stated that it was the obstetricians’ duty. Table 2 presents the pregnant women’s source of information and their preferences.

**Table 1.** Sociodemographic Data of the Pregnant Women at Al-Zahra Hospital

Variables	No. (%)
**Age (y) mean ± SD (min-max)**	30.39 ± 5.42 (18 - 43)
**Employment status**	
Homemaker	313 (84.8)
Employed	56 (15.2)
**Parity status**	
Nulliparous	141 (38.2)
Multiparous	228 (61.8)
**Residency**	
Urban	285 (77.2)
Rural	84 (22.8)
**Education level**	
Illiterate	7 (1.9)
Elementary-secondary school	71 (19.2)
High-school-high-school diploma	167 (45.3)
University degree	124 (33.6)
**Mode of previous delivery**	
Normal vaginal delivery (NVD)	157 (42.5)
Cesarian section	71 (19.2)
First pregnancy	141 (38.2)
**Pain intensity during the last NVD**	
Low	26 (16.6)
Medium	35 (22.3)
Intense	38 (24.2)
Very intense	58 (36.9)

**Table 2.** Pregnant Women’s Source of Information and the Preferred Ones

Variables	No. (%)
**Pregnant women’s information sources**	
Friends and relatives	123 (30.4)
Physicians	26 (6.4)
Nurses – midwives	27 (6.7)
Radio and television	41 (10.1)
Internet	170 (42)
Others	18 (4.4)
**Preferred sources**	
Educational brochures	21 (6.2)
Physicians	171 (50.1)
Nurses	34 (10)
Internet	96 (28.2)
Radio and television	12 (3.5)
Other	7 (2.1)

Moreover, 283 (83%) were ready to accept this method, 259 (76%) were willing to pay the expenses, and 294 (86.2%) requested this method. Besides, 166 (48.7%) had no fear of the procedure. Nonpharmacologic methods were the first choice for 164 (48.1%), while Entonox was the last choice for 26 (7.6%). The frequency of the respondents’ answers is presented in Table 3. 

A significant association was observed between maternal level of education and willingness to pay for PL (P = 0.006), knowledge of who performs it (P = 0.015), requesting a PL (P = 0.0001), options related to the preferred method for PL (P = 0.001), and being ready to pay for a PL service (P = 0.0001).

**Table 3.** The Frequency of Pregnant Women’s Answers

Variables	No. (%)
**Who will perform a painless delivery for you? **	
Obstetricians	218 (63.9)
Anesthesiologist	6 (1.8)
Nurse	0 (0)
Midwife	108 (31.7)
I do not know.	9 (2.6)
**Do you want a painless delivery?**	
Yes, I want a painless delivery.	283 (83)
No, I do not want a painless delivery.	39 (11.4)
I do not know.	19 (5.6)
**Are you afraid of painless delivery?**	
No, I am not afraid of painless delivery.	166 (48.7)
I am a little afraid of painless delivery.	78 (22.9)
I am moderately afraid of painless delivery.	24 (7)
I am very afraid of painless delivery.	73 (21.4)
**What is your preferred method for painless delivery?**	
Nonpharmacologic methods	164 (48.1)
Regional anesthesia	45 (13.2)
Entonox	26 (7.6)
Intravenous	76 (22.3)
I do not know.	30 (8.8)
**Are you ready to apply for a painless delivery service?**	
Yes, I am ready to request a painless delivery service.	294 (86.2)
No, I am not ready to request a painless delivery service.	35 (10.3)
I may be ready to request a painless delivery service.	12 (3.5)
**Are you ready to pay extra for a painless delivery?**	
Yes, I am ready to pay extra for a painless delivery.	259 (76)
No, I am not ready to pay extra for painless delivery.	69 (20.2)
I may be ready to pay extra for a painless delivery.	13 (3.8)

## 5. Discussion

Although the women’s knowledge was not satisfactory, their attitude toward the subject was completely positive. This finding could be very promising, given that obtaining informed consent is the first step for any medical intervention. The pregnant women’s acceptance rate of this method was very high and significant; they were ready to pay to receive this facility. Therefore, it is the responsibility of health policy-makers to provide awareness and accurate information for pregnant women and, more importantly, to create conditions so that they could benefit from this technique. Simply chanting the slogan of promoting NVD under PL is not enough, and reducing the rate of CS needs the provision of measures, without which this process will fail. Without providing the necessary conditions, threats and coercion on gynecologists and hospitals will not work either. In contrast, these one-dimensional measures endanger the lives of the mother and the fetus. Recently, several meetings have been held to address the challenges of PL. However, the main problem, which is the severe shortage of anesthesiologists and, thus, the absence of a responsible anesthesiologist for the process, still remains. In fact, as soon as a pregnant woman is scheduled for PL, the anesthesiologist should be involved. In this way, after obtaining a medical history and performing a physical examination, the optimal method of PL is chosen, and informed consent is obtained. Standard monitoring is used during the procedure until the birth, and the vital role of the anesthesiologist continues. In fact, the anesthesiologist is responsible for maintaining maternal hemodynamic stability from admission until delivery and performing any necessary interventions according to the neonates’ Apgar score. The fetus is continuously monitored during this process, and the anesthesia and surgery teams should be prepared if an emergency CS is needed.

A notable finding was that the source of information about PL was physicians in only 6.4% of the cases; more than half of the participants stated that it was necessary to receive information from physicians. Most of them thought that PL was performed by obstetricians, followed by midwives, and only 1.8% of them were aware that PL was performed by the anesthesiologist. Most women preferred nonpharmacologic methods, such as acupuncture, and had the least confidence in inhaling gases such as Entonox. Besides, a very small percentage preferred epidural anesthesia. As expected, mothers with higher education were more willing to accept this method, were more prepared to pay the costs, and had less fear and anxiety. Notably, the primary source of information was the internet, which should be corrected. The acquisition of information from the internet by nonspecialists with insufficient medical knowledge results in misinterpretation of the data. In addition, whether people receive information from reliable sources is debatable. Most respondents stated that it is necessary to receive information from physicians. A study in Egypt reported that most women had a negative attitude towards PL, and their information was very poor (12), which was in line with Moradi’s study in Kerman (Iran) and contrary to the current study. They found that 90.76% of the mothers did not accept this method. Nevertheless, most of them obtained their information via the internet, similar to our findings in Gilan, Iran (13). In the study by Pasha H in Iran, conducted in a university hospital, pregnant women’s awareness of PL, specifically of Entonox, was poor. This finding was consistent with that of the present study; however, their source of information was midwives, and they believed that it was physicians’ duty to give them accurate information and perform the procedure (14). A recent study in Turkey also showed that mothers’ level of knowledge of PL was not acceptable (15). Prakash A in Island concluded that maternal knowledge and acceptance of PL was poor; however, women with previous childbirth experience were significantly more inclined towards PL. Misinformation was one of the main reasons for not accepting PL (16). RV Shidhaye et al. in India reported that most of the participants still suffered from labor pain due to a lack of awareness about the availability of PL services (17). Studies conducted in developing countries have concluded that no structured planning is made to control pain based on the belief that childbirth is a physiological process, and this is the main reason for women’s lack of awareness and negative attitudes toward PL (10).

As mentioned, the findings of studies show discrepancies that can be explained by differences in the studied populations. Women’s sociodemographic status, beliefs, and culture, the medical facilities and economic status of society, and the importance that health policy-makers attach to this matter have all been influential factors that differ from region to region.

### 5.1. Limitations

Private centers were not included in this research, which can be a limitation of this study.

### 5.2. Suggestions

Considering the promising results regarding maternal attitude and acceptance, it is necessary to conduct more studies to solve the problem and remove the barriers.

### 5.3. Conclusions

Although the maternal knowledge status regarding the PL process was poor overall, the acceptance rate was promising. Most of the pregnant women were willing to receive PL. The important point was their source of information, which needs to be corrected. Medical teams must administer effective interventions to provide accurate information and resolve women’s doubts. Moreover, the principal health policy-makers should use correct and targeted strategies.

## Data Availability

The dataset presented in the study is available on request from the corresponding author during submission or after publication. The data are not publicly available due to privacy reasons.
